# Tailoring the wrap: intraoperative functional lumen imaging probe (FLIP) during hiatal hernia repair

**DOI:** 10.1007/s00464-024-10851-6

**Published:** 2024-05-09

**Authors:** Hemasat Alkhatib, AJ Haas, Ali M. Kara, Clara Lai, Alejandro Feria, Sergio Bardaro, Amelia Dorsey, Kevin El-Hayek

**Affiliations:** 1 Division of General Surgery, Department of Surgery, The MetroHealth System, 2500 MetroHealth Drive, Cleveland, OH 44109 USA; 2 Case Western Reserve University School of Medicine, 10900 Euclid Ave., Cleveland, OH 44106 USA

**Keywords:** FLIP technology, Hiatal hernia repair, Fundoplication, Refractory gastroesophageal reflux disease, Impedance planimetry, Distensibility index

## Abstract

**Introduction:**

The introduction of the functional lumen imaging probe (FLIP) has provided objective, real-time feedback on the geometric variations with each component of a hiatal hernia repair (HHR). The utility of this technology in altering intraoperative decision-making has been scarcely reported. Herein, we report a single-center series of intraoperative FLIP during HHR.

**Methods:**

A retrospective review of electronic medical records between 2020 and 2022 was conducted and all patients undergoing non-recurrent HHR with FLIP were queried. Patient and hernia characteristics, intraoperative FLIP values and changes in decision-making, as well as early post-operative outcomes were reported. Both diameter and distensibility index (DI) were measured at 40 ml and 50 ml balloon inflation after hiatal dissection, after hiatal closure, and after fundoplication when indicated.

**Results:**

Thirty-three patients met inclusion criteria. Mean age was 62 ± 14 years and mean BMI was 28 ± 6 kg/m^2^. The majority (53%) were type I hiatal hernias. The largest drop in DI occurred after hiatal closure, with minimal change seen after fundoplication (mean DI of 4.3 ± 2. after completion of HH dissection, vs 2.7 ± 1.2 after hiatal closure and 2.3 ± 1 after fundoplication when performed). In 13 (39%) of cases, FLIP values directly impacted intraoperative decision-making. Fundoplication was deferred in 4/13 (31%) patients, the wrap was loosened in 2/13 (15%); the type of fundoplication was altered to achieve adequate anti-reflux values in 2/13 (15%) patients, and in 1/13 (3%) the wrap was tightened.

**Conclusion:**

FLIP measurements can be used intraoperatively to guide decision-making and alter management plan based on objective values. Long-term outcomes and further prospective studies are required to better delineate the value of this technology.

**Supplementary Information:**

The online version contains supplementary material available at 10.1007/s00464-024-10851-6.

In patients with medically refractory gastroesophageal reflux disease with or without hiatal hernia, performing a minimally invasive fundoplication with hiatal hernia repair (HHR) when indicated provides excellent symptom control [1–4]. However, given the observed post-operative dysphagia and bloating reported by 20–30% of patients, different degrees of fundoplication were studied, with data suggesting similar improved symptom management in patient undergoing a 270° compared to 360° fundoplication with less post-operative dysphagia [5–7]. While these studies have certainly guided technical aspects of the operation, many of its steps remain reliant on subjective feedback dependent on surgeon experience and training.

The emergence of functional lumen imaging probe (FLIP) technology has introduced an objective real-time measurement to foregut surgery. This technology uses impedance planimetry sensors to evaluate sphincter geometry, providing real-time values regarding the diameter and distensibility [8–10]. Su et al. published their extensive experience using FLIP technology in foregut surgery where the majority of their patient population were those undergoing fundoplication [11]. Most importantly, values obtained by FLIP technology during fundoplication surgery were found to corelate with specific patient outcomes, with ideal values delineated from those studies [12, 13]. This allowed for opportunities to refine the operative plan and augment surgeon intraoperative decision-making.

The use of FLIP technology in providing tailored surgeries for individual patients has become a center focus at our institute. In this study, we aim to describe how the use of FLIP technology has influenced our intraoperative decision-making, and the outcomes thereafter.

## Materials and methods

### Study design and patient selection

The study was submitted and approved by the Institutional Review Board (IRB). This was a retrospective cohort review, and all data were acquired using electronic medical records. Our patient population included all patients undergoing non-recurrent HHR with or without fundoplication, with the use of FLIP technology intraoperatively. This study was performed at a safety net metropolitan anchor hospital with academic affiliations. The operations were performed by a one surgeon (KE) between 2020 and 2022. Included indications were refractory gastroesophageal reflux disease and symptomatic hiatal hernia. Patients with recurrent hiatal hernias were excluded. Two additional patients were excluded due to unreliable or absent intraoperative FLIP measurements. The STROBE statement checklist was used to present this study [14].

### Study variables and outcomes

Prior to the study period, the standard operation performed by the primary surgeon was a HHR with partial 270° (Toupet) fundoplication. Our investigative period commenced upon the initiation of FLIP technology utilization by the primary surgeon to alter intraoperative decision-making. Using Toupet fundoplication as the foundational operation, the primary outcome was an intraoperative change in decision-making based on FLIP values, and the description of the implemented changes. Secondary outcomes included changes in distensibility index (DI) across the steps of the procedure. The DI is a value obtained using FLIP technology which reflects the compliance of the esophagogastric junction (EGJ). The higher the DI, the more compliant the sphincter is, while low DI indicates a stiffer EGJ that is less compliant. A more detailed description of values obtained by the FLIP technology has been previously described [8]. Other outcomes were post-operative complications including dysphagia, bloating, persistent reflux requiring medications, oral intake intolerance, leak and vomiting. All post-operative symptoms were collected as binary yes/no inquires. These outcomes were measured at two time points: 30 day follow up, and 6 months follow up. This is the standard post-operative follow-up for all our patients. We also report on radiographic recurrence rates, as our standard of care post-operative follow up includes upper gastrointestinal series study at 6 months. Finally, we also collected demographic, patient and hernia characteristics. The hernia size was documented according to radiographic findings obtained from the preoperative timed esophagogram.

### Preoperative work up and operative technique

**Fig. 1:**
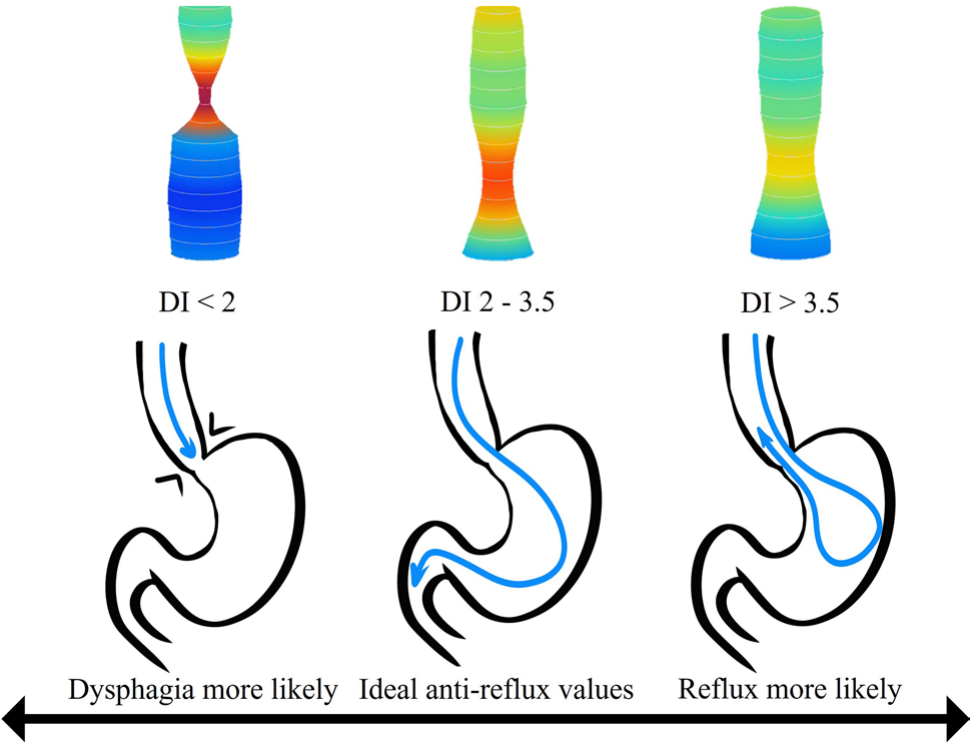
Illustration of different DI values that can be obtained using FLIP and their interpretations based on previously published data [12, 15]

**Fig. 2:**
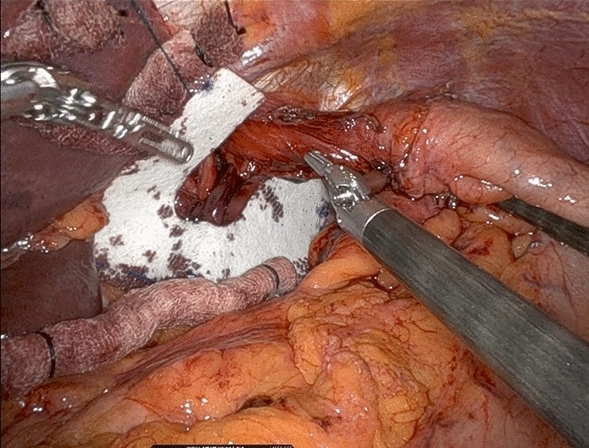
U configuration of hiatal mesh

Preoperative work up for patients undergoing this procedure was a timed esophagogram and an esophagogastroduodenoscopy. Manometry was not routinely obtained for our patient population and did not alter the wrap choice. Instead, all decisions regarding the wrap were made intraoperatively guided by FLIP values. All operations were performed robotically by a single surgeon (KE). The use of FLIP followed previously published expert consensus guidelines [8]. Briefly, measurements were obtained at the following timepoints (1) after hiatal dissection, (2) after hiatal closure, and (3) after fundoplication when performed. While FLIP values are obtained, the patient is kept in reverse Trendelenburg position and pneumoperitoneum is released. An 8 cm catheter is used, and values are obtained at balloon fill of 30 ml, 40 ml and 50 ml once values stabilize after approximately 30 s. Alteration in intraoperative plans were based on targeting DI values between 2 and 3.5, based on previously published studies correlating these values with good reflux symptom control with less post-operative complications [12, 15]. An illustration of these values and their interpretations based on previously published data are represented in Fig. 1. The hiatus is closed visually, and alterations (Tightening or loosening closure) are made in response to obtained DI values. When mesh is used, GORE^®^ BIO-A^®^ Tissue Reinforcement is used and placed in U configuration at the hiatus (Fig. 2). The use of mesh is typically reserved for patients with large Type III or IV hiatal hernia.

### Study analysis

Descriptive statistics were used to analyze the data, using frequencies, percentages, medians, interquartile ranges, means and standard deviation wherever appropriate. Missing data points, and patients lost to follow-up were reported.

## Results

### Demographics, hernia characteristics, and operative details

**Fig. 3:**
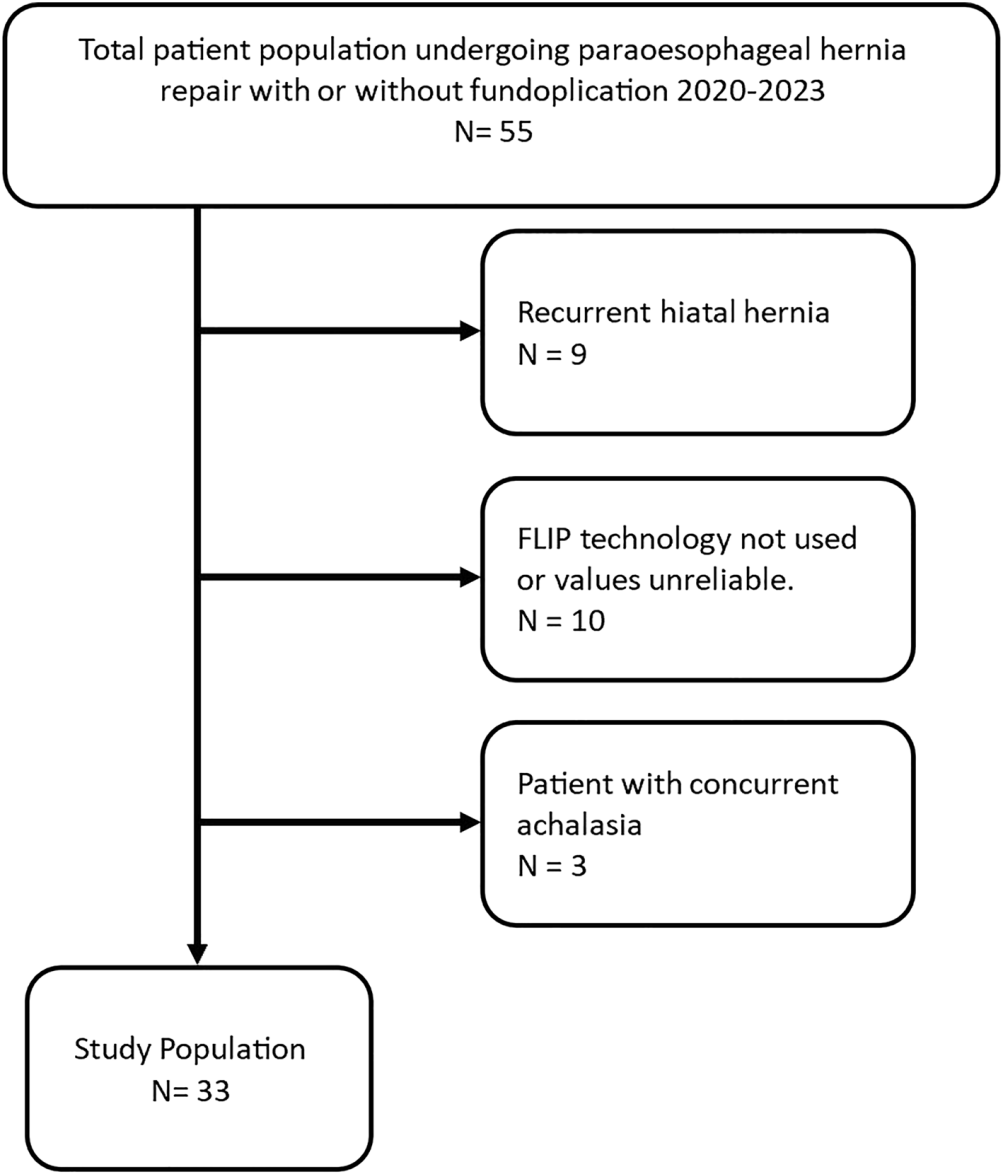
Population flowsheet

Figure 3 depicts the population enrollment process incorporating the inclusion and exclusion criteria. A total of 33 patients were included in the analysis. The demographics, hernia characteristics and operative details are presented in Table 1. The mean age of the cohort was 61.6 ± 14.0, and mean BMI was 28.2 ± 5.9. The majority were female (70%). The most common type of hiatal hernia was type I (55%) followed by type III (24%). The indication for repair was a symptomatic hiatal hernia for most of the population (94%) with more than half also reporting symptoms of reflux (58%). The most common fundoplication type performed was a 270° (Toupet) (82)%.

**Table 1:** Demographic, hernia characteristics and operative details

	(*n* = 33)
Age (mean ± SD)	61.6 ± 14.0
Gender (*n*, %)	
Female	10 (30%)
Male	23 (70%)
BMI (mean ± SD)	28.2 ± 5.9
Hiatal hernia type (*n*, %)	
I	18 (55%)
II	5 (15%)
III	8 (24%)
IV	2 (6%)
Indication for repair	
Symptomatic	31 (94%)
GERD	19 (58%)
OR time (mean ± SD)	163.4 min ± 33.1
Mesh used (*n*, %)	11 (33%)
Fundoplication type (*n*, %)	
Nissen	1 (3%)
Toupet	27 (82%)
Dor	1 (3%)
None	4 (12%)
Gastropexy (*n*, %)	20 (61%)
EBL (mean ± SD)	19 mL ± 16

### Primary outcomes

**Fig. 4:**
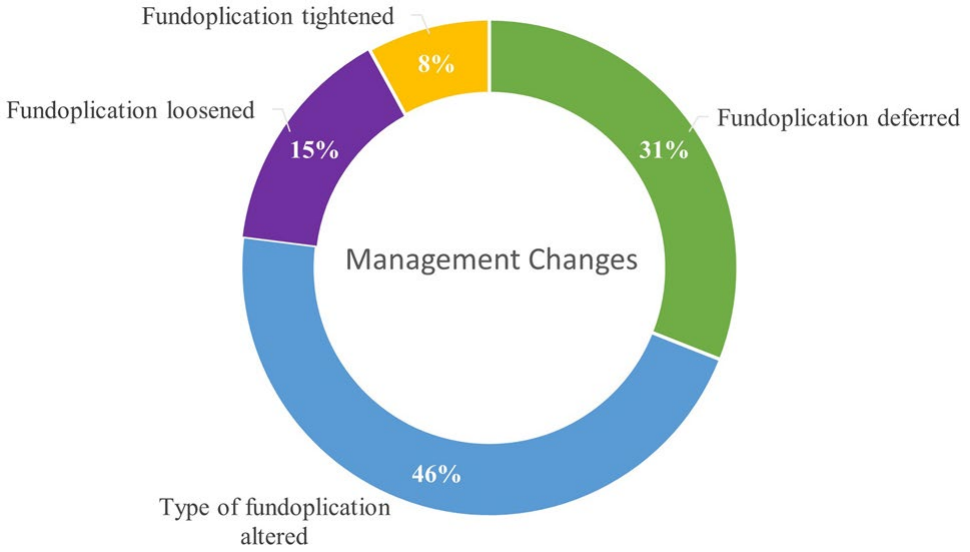
Management changes based on FLIP values

The use of FLIP technology altered intraoperative decision-making in 39% of the population. The management changes are summarized in Fig. 4. In 4/13 (31%) of patients, the fundoplication was completely deferred. In 6/13 (46%) of patients, the type of wrap was altered. In four of those patients, a floppier (180°) Toupet was done due to FLIP indicating adequate anti-reflux values with hiatal closure. In the remaining two patients in which the wrap was altered, the fundoplication was modified to a Nissen in one case and to a Dor fundoplication in the other. The decision to proceed to a Nissen was driven by no change in DI numbers despite adequate closure of the hiatus—assessed visually after further tightening (At 40 ml, DI of 5 at completion of hernia dissection, DI of 5 at conclusion of hiatal closure, and DI of 2 after completion of Nissen). A Dor fundoplication was chosen as the patient had DI value of 2 at the conclusion of the hiatal closure, and the surgeon wanted to avoid any further tightening of the EGJ while still providing a fundoplication flap. As the study period progressed, the surgeon was most likely to defer the fundoplication completely when obtaining adequate anti-reflux values rather than choosing a lesser degree wrap. The remaining patient population with intraoperative management changes comprised two patients in which the wrap was loosened, and one patient in which the wrap was tightened.

**Fig. 5:**
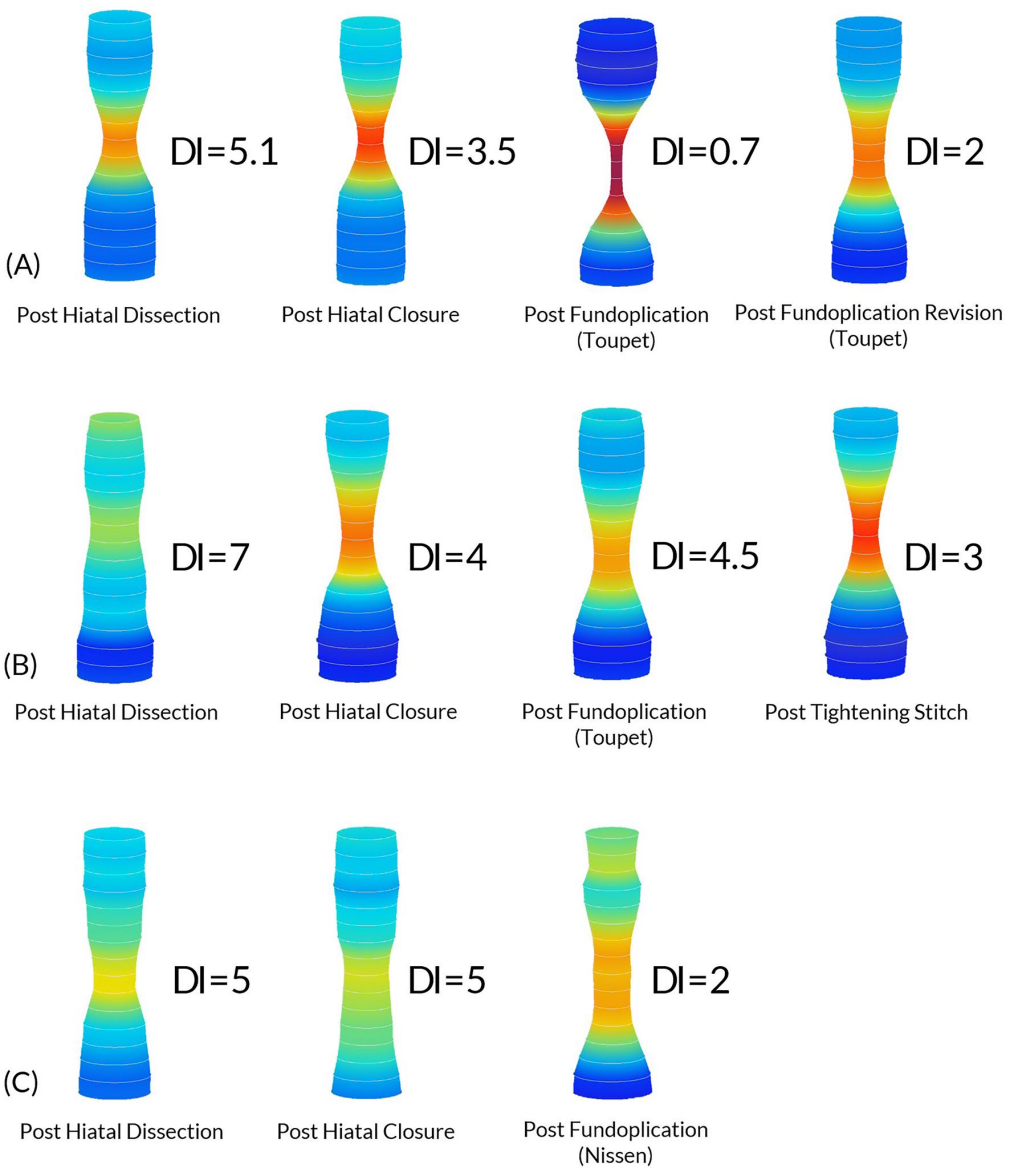
Illustration demonstrating dynamic changes to FLIP values intraoperatively in three patients (A, B and C), and the subsequent alteration in intraoperative management

Figure 5 illustrates three instances within our patient cohort in which FLIP technology was used to alter intraoperative surgical steps. Case (A): Post fundoplication DI values were significantly reduced, signaling potential EGJ constriction. Therefore, the fundoplication was revised to a more relaxed configuration resulting in DI values approximating target parameters. Case (B): Post fundoplication DI values remained above adequate anti-reflux parameters. Consequently, wrap tightening sutures were placed until DI values approached target levels. Case (C): the DI value did not change with hiatal closure, prompting the choice of a Nissen fundoplication instead of the standard Toupet. The supplemental video presentation also demonstrates how these intraoperative decisions are made in real-time.

### Secondary outcomes

**Fig. 6:**
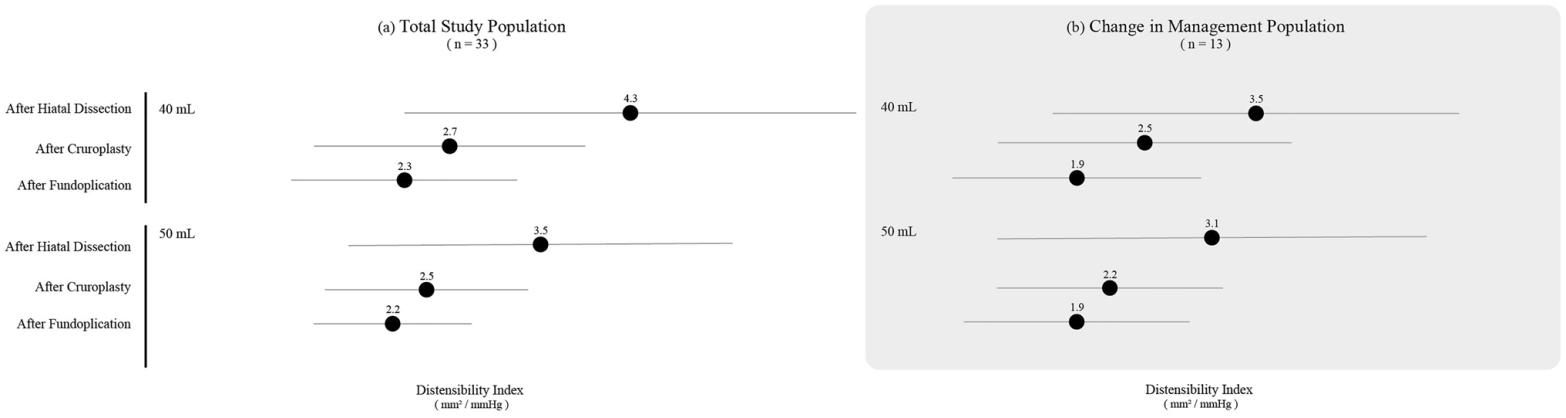
Distensibility index values **a** total population, **b** change in management population

Figure 6 depicts alterations in FLIP metrics across the entire cohort, as well as the subgroup patient population that underwent adjustments in intraoperative management. The most prominent reduction in DI values was evident subsequent to hiatal closure (DI at 40 ml 4.3 ± 2 after hiatal dissection to DI of 2.7 ± 1.2 after hiatal closure vs 2.3 ± 1 after fundoplication). The population that underwent management changes had overall lower DI scores at all measurement timepoints.

Table 2 demonstrates post-operative outcomes at 30 days and 6 months follow up. The majority of the population (32/33, 97%) completed 30 day follow up. By 6 months follow up, 49% were lost to follow up. The most common complaint was dysphagia at 25% at 30 days, and 29% at 6 months. There was one report of a contained post-operative leak, which was managed non-operatively with bowel rest and nasojejunal feeding with subsequent resolution without further ramifications.

**Table 2:** Post-operative outcomes at 30 days and 6 months

	(*n* = 32)	(*n* = 17)
Follow-up (mean ± SD)	Post-Op	Last
	(17.2 ± 6.2 days)	(7.4 ± 6 months)
Post-op complications		
Dysphagia	8 (25%)	5 (29%)
Bloating	3 (9%)	0
Persistent reflux	3 (9%)	1 (6%)
PO intolerance	3 (9%)	1 (6%)
Gastric dysmotility	0	0
Esophageal dysmotility	0	1 (6%)
SSI	0	0
Leak	1 (3%)	0
Vomiting	2 (6%)	1 (6%)

A total of 18 (55%) of patients completed radiographic follow up with a mean duration of 5 ± 3 months. Three radiographic recurrences (17%) were reported, though none were symptomatic or required reoperation. All three recurrences occurred in patients with Type III hiatal hernias that underwent HHR with mesh, and Toupet fundoplication. DI values measured at completion of the fundoplication, at 40 ml balloon fill for the three patients were 2.5, 2.2 and 2.3. One patient had a floppy 180° instead of a full 270° Toupet fundoplication done in response to FLIP values indicating adequate anti-reflux with hiatal closure.

## Discussion

The findings of our study contribute valuable insights to the existing literature concerning the utility of FLIP values in assisting surgeons’ intraoperative decision-making during anti-reflux surgery. Our study demonstrated the highest reported rate of intraoperative plan modification based on FLIP values, with the surgeon refining the surgical technique in 39% of patients. These changes ranged from tightening the fundoplication, altering its type, to forgoing it completely. This underscores the prospective significance of this novel technology in the future of hiatal hernia and anti-reflux surgery.

Data that was previously released by a different institution regarding the utilization of FLIP reported lower incidences of employing its measurements to alter management strategies. Initial findings reported a 23% alteration rate [11], succeeded by a subsequent publication reporting a 10.9% rate [16]. This discrepancy is probably attributed to the protocols developed at the institution, which mandated the choice of wrap based on FLIP values. The mere existence of these protocols underscores the broad potential applications of this technology.

This technology has also enhanced our understanding of the EGJ configuration and the impact of surgical interventions on these geometric measurements. We saw the most significant reduction in DI values post hiatal closure, suggesting potential reflux management without fundoplication. This aligns with findings from previous publications [16, 17], raising the crucial question: can restoring the EGJ’s anatomical position through hiatal closure alone effectively alleviate reflux symptoms and mitigate postoperative complications associated with fundoplication? While this question was assessed previously [18, 19], none of the modalities used allowed for intraoperative use that tailors surgical management.

The DI numbers in our patient population with intraoperative changes were slightly lower than the overall population. This is likely due to the changes most commonly being a response to lower DI values (i.e., deferring fundoplication, loosening the wrap). Upon closer analysis of the management modifications using FLIP within our dataset, a prominent trend emerges. As the study duration progressed, the prevailing alteration in management involved omitting fundoplication completely if sufficient anti-reflux values were achieved solely through hiatal closure. This shift likely mirrors the accumulation of experience with technology, fostering comfort in implementing more substantial adjustments. Our changes also encompassed tightening of the wrap and the selection of a higher degree wrap (Nissen) guided by FLIP values. These variances emphasize the diversity in EGJ geometry among patients and highlight the potential limitations of universally applying standardized surgical approaches.

Our study has several limitations. The small patient cohort and the absence of a comparative control population without the use of FLIP technology to alter intraoperative decision-making prevents meaningful comparisons and conclusions regarding outcomes of using this technology. One observation is a notable postoperative dysphagia rate, higher than reported in prior studies. This discrepancy may be attributed to our binary yes/no data collection method, lacking clinical significance assessment. Importantly, no patients needed interventions like dilation or reoperations for reported symptoms. Patient-reported outcomes using a GERD health-related quality of life "GERD-HRQL” [20], though used at our institute, was inconsistently documented prohibiting analysis. Furthermore, although it is our standard practice at our institute to discontinue proton pump inhibitors postoperatively, there was no specific documentation of this in the electronic medical records; hence, we omitted reporting this variable. If proton pump inhibitors were resumed by other caregiver teams, it is our protocol to thoroughly investigate the patients' symptoms, and no patient within this cohort required reoperation for recurrent reflux. A substantial portion of patients were lost to 6-month follow-up, reflecting many previous studies including patients at a safety net hospital. Finally, our study presents a single surgeon's experience within a single-center, limiting the generalizability of our findings.

In conclusion, the incorporation of FLIP technology represents a significant step toward objectively evaluating the anatomical dynamics of the EGJ during anti-reflux surgery, with the goal of aligning with predetermined benchmarks associated with improved patient outcomes. Our study outlines several approaches in tailoring the operation for each patient. Based on this small series, we have shown that deferring fundoplication can be performed safely with good short-term results. Our intention is to contribute to the progression of FLIP research to prospective research focused on investigating patient outcomes. Future studies will focus on long-term outcomes, particularly in those patients who did not undergo standard anti-reflux fundoplication.

### Supplementary Information

Below is the link to the electronic supplementary material.

Supplementary file4 (PDF 343 kb)

Supplementary file3 (DOCX 22 kb)

Supplementary file1 (MP4 100017 kb)
