# Supplementary material for: Tailoring the wrap: intraoperative functional lumen imaging probe (FLIP) during hiatal hernia repair
Source: Surg Endosc. 2024 May 9;38(6):3425–32. doi: 10.1007/s00464-024-10851-6 (PMC11133156; doi:10.1007/s00464-024-10851-6)
Supplement: Supplementary file 2 — Supplementary file3 (DOCX 22 kb) [file 464_2024_10851_MOESM2_ESM.docx]

| Study Population N=33 | | | | | | Change in Management Population N=13 | | | | | |
| --- | --- | --- | --- | --- | --- | --- | --- | --- | --- | --- | --- |
| After hiatal dissection | | | | | | After hiatal dissection | | | | | |
| 33 | Diameter at 40 | 12.8 ± 2.5 | 32 | DI at 40 | 4.3 ± 2 | 13 | Diameter at 40 | 12 ± 2.4 | 13 | DI at 40 | 3.5 ± 1.8 |
| 33 | Diameter at 50 | 14.6 ± 2.4 | 32 | DI at 50 | 3.5 ± 1.7 | 13 | Diameter at 50 | 13.9 ± 2.7 | 13 | DI at 50 | 3.1 ± 1.9 |
| After hiatal closure | | | | | | After hiatal closure | | | | | |
| 33 | Diameter at 40 | 10.3 ± 2.6 | 33 | DI at 40 | 2.7 ± 1.2 | 13 | Diameter at 40 | 9.9 ± 2.2 | 13 | DI at 40 | 2.5 ± 1.3 |
| 33 | Diameter at 50 | 13 ± 7 | 33 | DI at 50 | 2.5 ± 0.9 | 13 | Diameter at 50 | 14.5 ± 10.5 | 13 | DI at 50 | 2.2 ± 1 |
| After fundoplication | | | | | | After fundoplication | | | | | |
| 29 | Diameter at 40 | 9.5 ± 2.5 | 29 | DI at 40 | 2.3 ± 1 | 9 | Diameter at 40 | 8.4 ± 2.4 | 9 | DI at 40 | 1.9 ± 1.1 |
| 29 | Diameter at 50 | 11 ± 2.6 | 29 | DI at 50 | 2.2 ± 0.7 | 9 | Diameter at 50 | 9.9 ± 2.6 | 9 | DI at 50 | 1.9 ± 1 |
